# Study on the formation mechanism of the digital health information divide and capital-driven pathways among the older adults

**DOI:** 10.3389/fpubh.2026.1913689

**Published:** 2026-10-29

**Authors:** Xiaowen Yang, Biao Wang, Mai Xu, Haibo Chen, Li Huang, Haoxiang Wang, Wanchao Chen, Qinhui Fan, Xiaoning Yu, Shenzhong Zhang, Sipeng Shen, Huiling Pan

**Affiliations:** 1 Nanjing Medical University Library, Nanjing, China; 2 School of Pharmacy, Nanjing Medical University, Nanjing, China; 3 Department of Biostatistics, Center for Global Health, School of Public Health, Nanjing Medical University, Nanjing, China; 4 Institute of Science and Technology, Nanjing Medical University, Nanjing, China; 5 The Affiliated Taizhou People’s Hospital of Nanjing Medical University, Taizhou, China

**Keywords:** Bayesian network, digital health divide, digital inclusion, health information behavior, older adults, structural capital

## Abstract

**Background:**

Artificial intelligence and digital health services have expanded access to health information, yet older adults remain vulnerable to a digital health information divide due to unequal digital literacy, resource access, social support, and psychological readiness. However, the multi-capital mechanisms underlying this divide remain insufficiently explained.

**Objective:**

Grounded in structural poverty theory and Wilson’s information behavior model, this study examines how economic, cultural, social, political, and psychological capital shape older adults’ health information behavior and contribute to the digital health information divide.

**Methods:**

Questionnaire data were collected on older adults’ demographic characteristics, five forms of structural capital, and 13 information behavior variables. A Bayesian network model was used to identify inter-capital relationships, behavioral pathways, and key nodes within the information behavior chain.

**Results:**

Different forms of capital affected health information behavior through direct, indirect, and cross-capital pathways. Cultural capital occupied a central position, improving self-efficacy and information source evaluation while promoting active and continuous information seeking through the accumulation of social and political capital. Psychological capital occupied an intermediate position across multiple network connections involving capital and information behavior variables. Social and political capital provided interpersonal support and institutional safeguards, whereas economic capital mainly influenced access and channel diversification indirectly. Accordingly, governance should combine capital empowerment, pathway-based precision intervention, and institutionalized long-term support.

**Conclusion:**

This study constructs a network-based framework linking multi-capital, behavioral pathways, and divide risk, offering theoretical and practical guidance for improving older adults’ digital health literacy and reducing the health information divide.

## 1 Introduction

In an era marked by the rapid advancement of information technology, artificial intelligence (AI) is fundamentally reshaping service models in the global health sector. AI-driven health information services—such as intelligent health platforms, recommendation algorithms, virtual health assistants, and telemedicine—have not only enhanced the efficiency of information dissemination but also expanded the possibilities for health management (1). However, the benefits of these technological innovations have not been equally accessible to all demographic groups, particularly the older adults. Due to factors such as technological complexity, limited information accessibility, and insufficient digital literacy, older adults face pronounced challenges in acquiring and utilizing health information—a phenomenon referred to as the “digital health information divide” (2).

In recent years, China has been rapidly transitioning into a deeply aging society. By the end of 2022, the number of people aged 60 and above had exceeded 280 million, accounting for 19.8% of the total population (3). Concurrently, national policy frameworks such as the Healthy China 2030 Planning Outline and the 14th Five-Year Plan for Aging Work have repeatedly emphasized the importance of leveraging information technologies to enhance national health literacy and promote healthy aging (4). Nevertheless, older adults continue to encounter significant barriers in engaging with AI-enabled digital health technologies. These barriers are particularly acute in the context of unbalanced urban–rural development and unequal resource distribution, where older adult individuals face challenges not only in accessing such technologies but also in evaluating and applying complex health information effectively. These limitations hinder their capacity for self-health management and pose broader challenges to social equity and public health governance (5).

Globally, AI is increasingly regarded as a powerful tool for narrowing disparities in health resource distribution. At the same time, however, its rapid development has also deepened the existing technological divide (6). In response, several developed countries have initiated strategies such as age-friendly digital design, health information education, and policy interventions aimed at narrowing the digital health information gap among older populations (7). For example, some developers of intelligent health platforms have adopted more intuitive user interfaces tailored to the needs of older users, providing personalized health recommendations (1, 8); governments and community organizations have implemented digital skills training programs to reduce technological anxiety and enhance information acquisition capabilities among seniors (9). In contrast, China has yet to establish a comprehensive strategy specifically targeting the older population in the context of AI-driven health services. There remains an urgent need to improve both the technological adaptability and usage capacity of older adults (10).

Against this backdrop, the present study seeks to address the following key questions: (1) How do older adults’ economic, cultural, social, political, and psychological capital influence their performance in different stages of health information behavior, and potentially affect their risk of experiencing an information divide? (2) How to formulate targeted strategies for bridging this divide? By exploring these questions, this study aims to contribute both theoretically and practically to the promotion of equitable access to health information and the construction of an intelligent and inclusive health service system.

## 2 Literature review: domestic and international research

Over the past 5 years, international research on the health information divide among the older adults has primarily focused on the behavioral characteristics of older adults in acquiring health information and the consequences of the digital divide (11). Overall, studies have found that while older adults are increasingly accessing health information through diverse media—including the internet—rather than relying solely on face-to-face consultations with physicians, significant disparities in access and utilization persist. For instance, surveys indicate that older individuals still place the greatest trust in and reliance on health information provided by healthcare professionals (e.g., doctors and pharmacists), followed by information from family and friends, whereas trust in online health information remains relatively low (12). Many older adults lack the confidence to assess the quality of online health information (12). This population faces multiple barriers to effective information use, including cognitive and skill limitations at the individual level, inadequate social support, and non-age-friendly technology design (11). A systematic review of 75 related studies identified 10 major types of health information commonly sought online by older adults, along with six main categories of internet-based channels. The influencing factors were classified into two broad groups: individual-level factors (e.g., demographic characteristics, health beliefs, digital skills, and self-efficacy) and source-related factors (e.g., credibility, usefulness, and ease of use of the information) (11). Furthermore, the barriers faced by older users in online health information acquisition can be grouped into 11 subcategories across three dimensions—personal, social, and technological. These include issues such as declining visual and auditory function, low health and digital literacy, anxiety or resistance toward technology, lack of family support, and the absence of age-appropriate devices and interfaces (11). Such barriers have contributed to the further marginalization of certain older populations in the digital age, exacerbating the phenomenon wherein “the information-rich become richer, while the information-poor become increasingly disadvantaged” (13). Additionally, some studies have quantitatively examined the relationship between health literacy disparities and the use of online health information. Findings show that only about 9.7% of older adults with limited health literacy use the internet for health-related purposes, compared to 31.9% of those with sufficient health literacy. This “literacy divide” remains statistically significant even after controlling for demographic and health status variables (14). These findings suggest that the digital health information divide is often intertwined with structural factors such as education level and health cognition (14). In response to these challenges, recent years have witnessed emerging digital interventions targeting older adults. For example, community-based digital health information workshops have been launched to enhance seniors’ internet skills and boost their confidence in using digital tools. Several intervention trials have reported reductions in computer anxiety and improvements in self-efficacy among participants following such training programs (11).

By the end of 2023, China’s older population aged 60 and above had reached 297 million, accounting for 21.1% of the total population (15). As the country undergoes concurrent processes of population aging and digital transformation, increasing scholarly and policy attention has been paid to the issue of the “health information divide among older adults” (16). In the Chinese academic context, this phenomenon is often conceptualized using terms such as “health information poverty” or “digital poverty,” which emphasize the barriers and deficiencies older individuals face in accessing, understanding, and utilizing health information (17, 18). Studies have shown that many older adults still rely primarily on traditional media and offline channels to obtain health knowledge and often demonstrate limited competence in engaging with new digital media, resulting in lagging information access, processing, and utilization relative to the societal average (19). Drawing from the framework of multidimensional poverty, some scholars have proposed that the health information divide among older adults can be understood as a form of multidimensional information deprivation. For instance, Li Jing et al., based on the Capability Approach, categorized this deprivation into two types: “informational capability poverty” and “informational functional poverty.” Using national survey data, their study found significant correlations between older individuals’ limitations in information access, acquisition, use, and perception, and their inability to effectively apply health information in practice (i.e., functional poverty) (20). These findings indicate that older adults in China continue to face substantial deficits in basic information-seeking skills and access to reliable information channels—factors that directly hinder their capacity to translate health information into informed decisions and behaviors (21, 22). Another stream of research has focused on the specific manifestations and causes of the digital divide among older Chinese citizens. This literature identifies three primary forms of digital poverty: lack of internet access, limited application of digital tools, and insufficient digital literacy (23, 24). These challenges stem from both internal and external factors. On the one hand, psychological barriers such as technological anxiety, concerns over online security, and resistance to digital adoption limit older adults’ engagement with emerging media (25, 26). On the other hand, structural issues—including the lack of age-friendly design in digital products and services, insufficient consideration of older adults’ needs in the promotion of intelligent technologies, and underdeveloped social support systems—further exacerbate digital exclusion among this population (27–29). Empirical findings from China echo international patterns: older adults with lower levels of education and income, as well as those residing in rural areas, are disproportionately disadvantaged in terms of access to and utilization of health information. The digital divide between urban and rural areas and across different regions remains pronounced. In response to these challenges, Chinese scholars have begun to explore potential interventions. Commonly proposed strategies include enhancing digital and health literacy among the older adults, improving the delivery of community-based health information services, and strengthening both family and societal support networks. These efforts are widely regarded as essential pathways toward narrowing the health information divide for older adults (30–33).

Current research on the digital health information divide among older adults reveals several limitations at the theoretical, methodological, and policy levels. Theoretically, macro-level structural factors and micro-level behavioral factors remain insufficiently integrated, resulting in fragmented findings and a lack of an integrative framework for explaining the relationships between structural barriers and psychological and behavioral factors in older adults’ information acquisition. Methodologically, most studies rely on cross-sectional data, which limits causal inference. Samples are often concentrated among urban, relatively well-educated older adults, with insufficient representation of rural residents, the oldest-old, and those with lower educational attainment. Some qualitative studies involve small samples, while interdisciplinary and cross-cultural comparisons remain limited. In policy and practice, digital health interventions are still at an exploratory stage, with limited diversity in intervention approaches and insufficient evaluation of policy instruments such as public information services and age-friendly media design. Some governance recommendations in the Chinese literature remain largely conceptual and lack empirical support, limiting their capacity to address the specific needs of different groups of older adults. Overall, further efforts are needed to integrate structural and behavioral perspectives, strengthen the explanatory scope and applicability of research evidence, and translate theoretical insights into policy practice while evaluating their effectiveness.

In response to these limitations, this study primarily addresses the insufficient integration of structural resources and individual information behavior by combining a multidimensional capital framework informed by the Theory of Structural Poverty with Wilson’s Information Behavior Model. It examines the associations between different forms of capital and various components of older adults’ health information behavior and uses these findings to inform targeted support strategies. The theoretical premise is that differences in health information behavior may be associated with multiple forms of capital and their interrelationships, which cannot be adequately understood by examining individual resource factors in isolation. Accordingly, this study includes economic, cultural, social, psychological, and political capital, together with information behavior variables derived from Wilson’s model, and employs correlation analysis and Bayesian network modeling to explore their associations and conditional dependencies. Online surveys are combined with offline surveys in community settings to broaden participation among older adults with varying levels of digital access, although this approach does not ensure adequate representation of disadvantaged groups. As the study remains cross-sectional and does not evaluate intervention effectiveness, its contribution lies in providing integrated evidence on resource–behavior associations and empirical insights to inform governance strategies. Causal relationships, the effectiveness of these strategies, and their applicability across population groups require further investigation through longitudinal, intervention, and comparative studies.

## 3 Theoretical framework

### 3.1 A multidimensional capital framework from the perspective of structural poverty

This study adopts the perspective of the Theory of Structural Poverty, which situates individual resource deprivation within broader social structures, institutional arrangements, and inequalities in the distribution of resources and opportunities (34, 35). Accordingly, older adults’ difficulties in acquiring and utilizing digital health information are examined in relation to their social and institutional contexts. Drawing on capital theory and related research, this study develops a multidimensional analytical framework encompassing economic, cultural, social, psychological, and political capital. These five forms of capital constitute a theoretical integration tailored to the research questions, rather than a unified classification inherent in the Theory of Structural Poverty.

The conceptualization of economic, cultural, and social capital draws primarily on Bourdieu’s account of capital. Bourdieu emphasizes that capital requires accumulation over time, that different forms of capital can be converted into one another under specific conditions, and that their unequal distribution is closely associated with differences in access to social opportunities. Economic capital primarily comprises economic resources available to individuals; cultural capital includes enduring cultural dispositions, cultural goods, and institutionalized forms such as educational qualifications; and social capital refers to actual or potential resources accessible through relatively durable networks of relationships (36). Accordingly, this study considers the material conditions provided by economic resources, the cultural resources accumulated through education and learning, and the support available through social relationships, and explores their associations with older adults’ digital health information behavior. Cultural capital is not synonymous with all individual skills, nor is social capital equivalent to interpersonal contact or social participation alone. The interpretation of their indicators therefore requires attention to the resources these indicators represent.

The inclusion of psychological capital draws on research on positive psychological capital. Luthans et al. identify self-efficacy, hope, optimism, and resilience as its principal components, emphasizing their combined contribution to a higher-order construct and its relationship with performance (37, 38). Drawing on this resource-oriented perspective, the present study incorporates psychological capital to examine the broad psychological resources that older adults can mobilize when coping with digital technologies and information-related challenges. In this study, psychological capital is operationalized through general self-efficacy, emotional regulation ability, psychological resilience, and emotional stability. General self-efficacy here refers to broad confidence in coping with difficulties across situations, whereas information behavior self-efficacy, included separately under Wilson’s model, refers specifically to confidence in searching for, evaluating, and using digital health information. The two constructs are therefore conceptually related but operate at different levels: the former represents a general psychological resource, while the latter represents confidence in performing specific information-related tasks. Because the indicators used in this study differ from the established four-component conceptualization of psychological capital, interpretation of this variable is restricted to the psychological resources actually assessed and does not imply comprehensive measurement of the established construct.

The inclusion of political capital is informed by sustainable livelihoods research addressing power relations, institutional participation, and resource access. This literature suggests that opportunities to access resources depend not only on their availability but also on individuals’ positions within institutional relationships and their capacity to participate and assert their rights (39). Accordingly, this study examines older adults’ resource-related connections with public institutions through perceived access to public resources, sense of political participation, and perceived fairness in resource distribution. Political and social capital differ in their analytical emphasis: the former concerns participation and resource access within institutional relationships, whereas the latter concerns resources available through social networks. Nevertheless, the political capital indicators used in this study primarily capture subjective experiences. They should not be equated with actual political influence or treated as substitutes for objective measures of institutional provision.

Together, these five forms of capital provide complementary perspectives on older adults’ digital health information circumstances through material, cultural, relational, psychological, and institution-related resources. Their joint inclusion does not imply identical theoretical origins, processes of formation, or levels of analysis. This study focuses on resources that individuals possess or can mobilize and on their relationships with information behavior. Individual characteristics describe personal attributes; skills concern the execution of specific tasks; capabilities concern the conditions for undertaking action; and institutional resources refer to external provision and support. Although these concepts may overlap with capital, they are not interchangeable. The inclusion of a particular characteristic or capability as an indicator of capital therefore requires justification in relation to its development, significance as a resource, and questionnaire item content.

Within this framework, the five forms of capital are treated as complementary resource dimensions rather than as a unified taxonomy derived entirely from Bourdieu. Economic, cultural, and social capital provide the Bourdieusian foundation of the framework, representing material resources, resources accumulated through education and learning, and resources accessible through social networks, respectively. Psychological and political capital extend this foundation by capturing two additional resource domains relevant to older adults’ digital health information behavior: mobilizable psychological resources for coping with information-related challenges and perceived access to institutional resources and opportunities for participation. The Theory of Structural Poverty provides the overarching perspective for examining how inequalities in the distribution of these material, cultural, relational, psychological, and institution-related resources are associated with differences in information behavior. The five dimensions have distinct theoretical origins and operational meanings but are jointly included because each represents a type of resource that individuals possess or can mobilize. Their integration therefore provides a multidimensional resource perspective for examining the digital health information divide among older adults.

### 3.2 Wilson’s information behavior model

Wilson’s Information Behavior Model is a foundational theoretical framework in the field of information science, widely employed to study individual behaviors and influencing factors in the processes of information need, seeking, evaluation, and use. The model emphasizes that information behavior is driven by individual needs, but its occurrence and development are influenced by multiple factors, including personal psychological characteristics, social context, and the information environment (40, 41), as shown in Figure 1. Wilson’s model proposes a hierarchical pathway of information behavior. When individuals encounter an information need, they initially engage in the process of information acquisition, which may occur either through active searching or passive encountering. Subsequently, individuals evaluate the information obtained to assess its credibility and utility. Finally, based on the evaluated information, they make decisions or take corresponding actions. One of the model’s key contributions is its conceptualization of information behavior as more than a purely cognitive activity. It acknowledges the joint influence of internal individual factors—such as motivation, anxiety, and cognitive load—and external environmental factors—such as social support and information accessibility. Furthermore, Wilson’s model integrates concepts from related theories, including the Stress and Coping Theory and various Information Seeking Theories, enabling a more comprehensive explanation of the complexity of information behavior. In recent years, the model has been increasingly applied in the field of health information research, providing analytical tools for understanding how different populations seek and utilize health information. These applications have yielded valuable insights for optimizing health information service systems and tailoring interventions to specific user needs (42).

**Figure 1:**
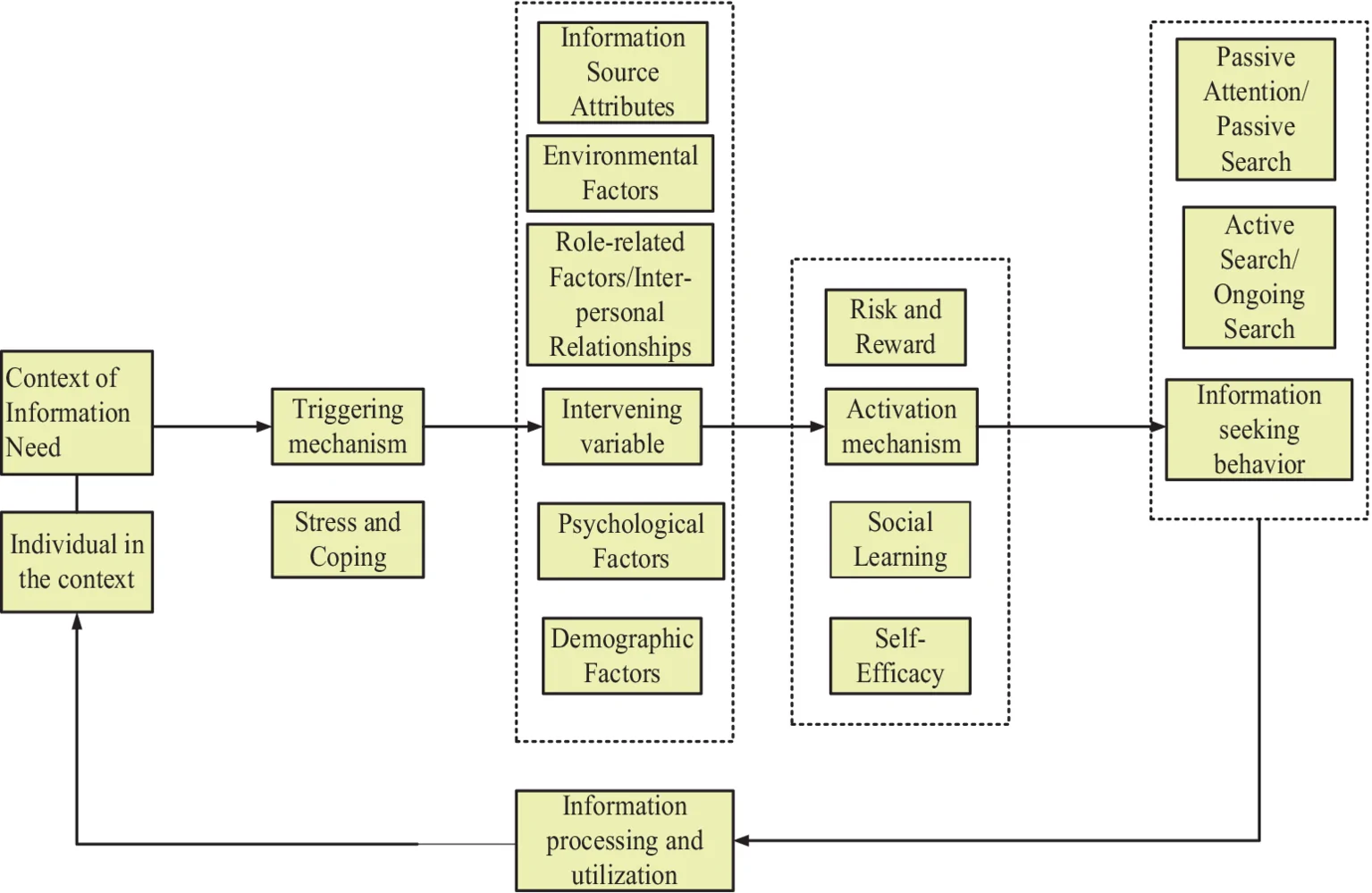
Wilson’s information seeking behavior model proposed in 1996.

## 4 Theoretical integration and research implementation

This study integrates a multidimensional capital framework informed by the Theory of Structural Poverty with Wilson’s Information Behavior Model. The former focuses on the resources older adults possess or can mobilize and differences in their distribution, whereas the latter provides a framework for organizing the context of information needs, activating mechanisms, intervening variables, and different modes of information seeking. Wilson’s model emphasizes that information needs do not necessarily translate into information seeking; psychological, role-related, interpersonal, and environmental factors, together with information source attributes, may facilitate or inhibit this process (37, 38). Integrating these perspectives enables this study to examine the relationships between resource conditions and information behavior.

The integrated framework comprises two interrelated analytical levels. The resource level encompasses economic, cultural, social, psychological, and political capital, describing participants’ resource conditions. The information behavior level encompasses the context of information needs, relevant psychological and environmental factors, and information-seeking behaviors organized according to Wilson’s model, describing different aspects of participants’ engagement with health information. Accordingly, this study explores associations and conditional dependencies both among the forms of capital and between capital and information behavior variables, without presupposing that all forms of capital relate to information behavior through the same pathways.

The distinction between these analytical levels does not imply that their constructs are inherently mutually exclusive. For example, psychological capital and information-seeking self-efficacy may share psychological content, while social capital and interpersonal relationship variables may capture related relational resources. Theoretical integration therefore requires attention to both conceptual connections and measurement boundaries. The inclusion of variables derived from different theoretical frameworks does not, in itself, establish adequate discriminant validity. Furthermore, this study primarily uses individual-level survey data. The structural poverty perspective guides the interpretation of resource inequalities but does not imply that the effects of macro-level social structures are directly measured or tested.

In implementing this framework, questionnaire data on capital and information behavior are analyzed using correlation analysis and Bayesian network modeling to explore relationships among variables. Given the cross-sectional design, the resulting network represents statistical dependencies within the sample and informs hypotheses for subsequent research. Directed edges in the network are not treated as direct evidence of temporal ordering, causal effects, or mediation mechanisms.

### 4.1 Questionnaire design and variable measurement

To systematically explore the behavioral characteristics and influencing factors related to older adults’ acquisition and use of digital health information, this study developed a survey questionnaire based on the theoretical integration of the Theory of Structural Poverty and Wilson’s Information Behavior Model. The questionnaire serves as the primary data collection instrument for the subsequent empirical analysis. Its design closely follows the dimensional structure and logical framework of the theoretical models, ensuring systematic measurement of all relevant latent variables. The questionnaire used in this study is available in Supplementary file 1 (Questionnaire). The questionnaire items were developed by the authors based on the theoretical frameworks and variable definitions adopted in this study, with wording tailored to older adults’ digital health information behavior. They were not taken from or adapted from existing standardized scales. The correspondence between questionnaire items and the 18 study variables, together with summaries of item content, is provided in Supplementary Table S1.

Within the multidimensional capital framework informed by the Theory of Structural Poverty, questionnaire indicators were specified across five dimensions: economic, cultural, social, psychological, and political capital. Economic capital was measured through monthly income, fixed asset ownership, consumption expenditure levels, and perceived economic stress. Cultural capital was assessed using four indicators: highest educational attainment, frequency of cultural participation, self-reported proficiency in searching for and acquiring information online, and frequency of ongoing learning and knowledge updating (Items 46–49). Educational attainment reflects institutionalized educational experience, whereas cultural participation and ongoing learning reflect practices through which individuals engage with cultural resources and update their knowledge. Internet information search and acquisition skills were included to capture information acquisition capabilities in digital contexts. Given their conceptual overlap with digital competencies, these indicators constitute a context-specific operationalization of cultural capital rather than a comprehensive measure of the construct. Theoretically, educational and cultural learning resources may facilitate the understanding of health information, while information search skills may support access to relevant resources. Social capital was assessed using four indicators: supportive social networks, support available when facing difficulties, frequency of community participation, and interpersonal interaction (Items 50–53). The first two indicators concern whether individuals have stable networks capable of providing assistance and can obtain support through these networks; the latter two reflect social participation and relationship maintenance. This dimension therefore captures both resources available through social networks and associated participatory practices, without equating participation or interaction frequency alone with social capital. These items assess social resources in everyday life. Their theoretical relevance to health information behavior lies in the possibility that accessible social networks provide information leads, practical assistance, and support for understanding information; however, health information support was not directly measured by these items. Psychological capital was measured through self-efficacy, emotional regulation ability, psychological resilience, and emotional stability, whereas political capital was examined through perceived access to public resources, perceived participation in social and political decision-making, and perceived fairness in resource distribution.

For the health information behavior component, the questionnaire was designed in alignment with the full structure of Wilson’s Information Behavior Model, with items corresponding to each theoretical element. These included: Contextual identification of information needs; Information-seeking motivations under the Stress and Coping Theory; Individual psychological traits (e.g., anxiety level, self-confidence); Demographic background variables (e.g., age, gender, education); Role-related factors (e.g., role in family health decision-making); Interpersonal networks (e.g., influence of friends and family on information behavior); External environmental factors (e.g., accessibility of health resources in residential area); Source characteristics (e.g., perceived credibility and usability of information); Information-seeking decision logic guided by Risk–Reward Theory; Imitation tendencies in information behavior influenced by Social Learning Theory; Confidence in information seeking as informed by Self-Efficacy Theory; Behavioral features such as passive attention and passive querying, active querying, and ongoing search.

Psychological capital and information behavior self-efficacy were treated as conceptually related but operationally distinct variables. Psychological capital refers to relatively broad psychological resources that older adults may mobilize across challenging situations, including general confidence in coping with difficulties, emotional regulation, psychological resilience, and emotional stability. Information behavior self-efficacy, by contrast, is task-specific and refers to confidence in one’s ability to search for, evaluate, and use digital health information. Thus, the former was defined at the level of general psychological resources, whereas the latter was defined at the level of a specific information-related task. Statistically, the two variables were constructed from non-overlapping questionnaire items, scored independently, and entered into the correlation analysis and Bayesian network as separate variables. However, because no dedicated discriminant validity test was conducted between these two author-developed measures, their statistical separation should be understood as an operational distinction rather than definitive evidence of discriminant validity, and some residual conceptual overlap cannot be excluded.

Each latent variable was measured using 3–4 items rated on a 5-point Likert scale (1 = strongly disagree, 5 = strongly agree), ensuring continuous data suitable for statistical analysis. The questionnaire was designed with clear and concise language, avoiding complex terminology to enhance comprehension and response willingness among older participants. To ensure the content validity and scientific rigor of the instrument, a pilot test was conducted prior to formal administration. Based on respondent feedback, revisions were made to item phrasing and question order to improve clarity and coherence. The final version of the questionnaire received ethical approval from the Nanjing Medical University Ethics Committee [Approval No. NMU-EC (2025)-6], and the design and implementation adhered strictly to ethical standards for scientific research. Reliability analysis showed that the Cronbach’s alpha coefficients for all latent variables exceeded 0.7, indicating good internal consistency. The Kaiser–Meyer–Olkin (KMO) value was 0.825, and Bartlett’s test of sphericity was statistically significant (*p* < 0.001), confirming the suitability of the data for factor analysis.

### 4.2 Data collection and preprocessing

Participants aged 60 years and above were recruited using non-probability convenience sampling through both online and offline surveys. The online survey was distributed via the Wenjuanxing platform, while the offline component was administered by trained surveyors through in-person interviews at community health service centers, universities for the older adults, and senior activity centers. Offline recruitment provided an opportunity for older adults with limited digital access to participate without completing an online questionnaire. However, this approach did not ensure representativeness of the broader older population; individuals with limited digital access, lower educational attainment, or limited participation in community activities may have been underrepresented. Before analysis, all questionnaires were screened for completeness, logical consistency, and completion time. Of the 541 questionnaires collected, six were excluded because they were incomplete. The remaining 535 questionnaires contained complete data for all variables included in the analysis; therefore, no missing-data imputation was performed. Logical consistency and completion time were examined as quality-control indicators, but the six actual exclusions were all due to incomplete responses.

### 4.3 Data analysis

Following data collection and preprocessing, the means and standard deviations (SD) were computed for the five dimensions of structural poverty and the 13 dimensions of Wilson’s information behavior model. Spearman rank correlation coefficient and Bayesian network modeling were applied to analyze them.

#### 4.3.1 Spearman rank correlation coefficient

The Spearman rank correlation coefficient ( $r_{s}$ ) is a non-parametric statistical method used to assess the strength and direction of the association between two variables, which is defined as Pearson correlation coefficient between two ranked random variables. $r_{s}$ takes a value from −1 to +1 (43). A larger absolute value of the coefficient indicates a stronger correlation between the variables. For a data sample of size n, the raw data scores $A_{i}$ and $B_{i}$ are converted to ranks $\mathrm{rg}A_{i}$ and $\mathrm{rg}B_{i}$ , and $r_{s}$ is calculated as


$$r_{s}=1-\frac{6\sum d_{i}^{2}}{n(n^{2}-1)}$$

where $d_{i}=\mathrm{rg}A_{i}-\mathrm{rg}B_{i}$ is the significant difference between the two ranked variables.

To reduce false positives in multiple comparisons, this study employed the Bonferroni correction by adjusting the significance threshold to α/k , where *k* denotes the total number of statistical tests conducted.

#### 4.3.2 Bayesian network

The Bayesian Network (BN) is a probabilistic graphical model that integrates graph theory with Bayesian probability to represent the conditional dependencies among variables. Formally, a BN is represented as a directed acyclic graph (DAG), in which each node corresponds to a system variable and each directed edge encodes a probabilistic dependency between variables (44). This modeling framework is highly adaptable to various conditions, making it highly suitable for addressing complex problem scenarios.

For the discrete Bayesian network analysis, the summed score for each questionnaire dimension was divided by the number of items in that dimension, producing an average score ranging from 1 to 5 and placing all dimensions on the same scale. These average scores were then categorized using predefined cutoffs: low, 1.00–2.50; medium, >2.50–3.50; and high, >3.50–5.00. The cutoffs of 2.5 and 3.5 represent the midpoints between adjacent Likert response categories, namely between 2 and 3 and between 3 and 4, respectively, and were used to distinguish relatively low, medium, and high levels of each dimension. Discretizing the average scores into three states facilitates the estimation and interpretation of conditional probability relationships in the discrete Bayesian network. However, this conversion may result in some loss of information because differences among individuals within the same category are not fully retained. Therefore, discretization may affect the estimated relationships in the Bayesian network to some extent. Structural learning of the Bayesian network was performed using the tabu search algorithm implemented in the *bnlearn* R package, with the Bayesian Information Criterion (BIC) used as the scoring criterion. A total of 200 bootstrap samples were used to assess the stability of the network structure, and arcs with strength values greater than 0.75 were retained in the final network. In Figure 2, thicker arrows indicate greater relative arc strength.

**Figure 3:**
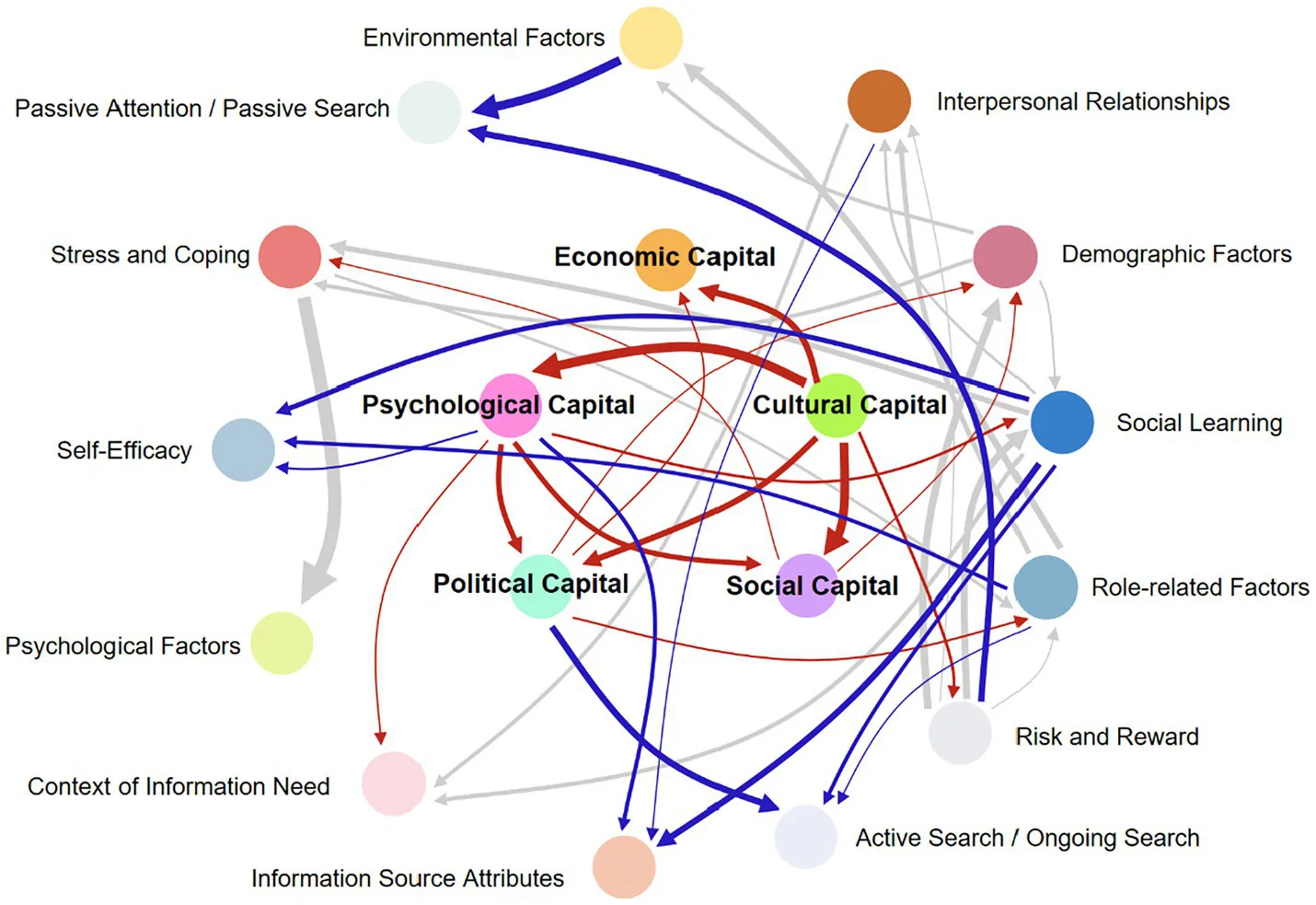
Integrated Bayesian network illustrating conditional-dependency relationships between structural capital, health information behavior, and behavioral proxy indicators relevant to the digital health information divide.

## 5 Results

### 5.1 Descriptive statistics analysis

This study conducted a descriptive statistical analysis of the five dimensions of structural capital and various components of information behavior. The results are presented in Table 1. The mean scores of the five types of structural capital ranged from 11.31 to 14.24, with standard deviations between 3.11 and 3.96, indicating a moderate degree of individual variation among the older respondents in terms of structural capital.

**Table 1:** Descriptive statistics of capital and information behavior variables.

Variables	$\bar{x}\pm \mathrm{SD}$
Economic capital	11.31 ± 3.68
Cultural capital	11.67 ± 3.45
Social capital	11.93 ± 3.96
Psychological capital	14.24 ± 3.11
Political capital	11.74 ± 3.74
Context of information need	12.00 ± 2.75
Stress and coping	11.84 ± 2.51
Psychological factors	10.17 ± 2.24
Demographic factors	9.84 ± 2.40
Role-related factors	9.71 ± 2.85
Interpersonal relationships	9.87 ± 2.53
Environmental factors	8.32 ± 2.75
Information source attributes	10.95 ± 2.60
Risk and reward	10.19 ± 2.35
Social learning	10.74 ± 2.46
Self-efficacy	10.61 ± 2.43
Passive attention/passive search	12.01 ± 3.32
Active search/ongoing search	12.75 ± 3.54

Among the five types of capital, psychological capital scored the highest ( $\bar{x}$ =14.24, SD = 3.11), suggesting that most older adults exhibit strong psychological attributes such as self-confidence, adaptability, and emotional regulation. These traits provide a foundational psychological drive for engaging in digital health information-seeking behavior. In contrast, economic capital ( $\bar{x}$ =11.31, SD = 3.68) and political capital ( $\bar{x}$ =11.74, SD = 3.74) received relatively lower scores, reflecting substantial constraints in material resources and institutional support among some older individuals. These may represent significant external barriers to their information access capacity.

With regard to the 13 dimensions of Wilson’s Information Behavior Model, the average scores ranged from 8.32 to 12.75, with standard deviations generally between 2.24 and 3.54, further indicating inter-individual variability. Higher scores were observed in dimensions such as Active Search/Sustained Inquiry ( $\bar{x}$ =12.75, SD = 3.54), Passive Attention/Search ( $\bar{x}$ =12.01, SD = 3.32), and Information Need Situations ( $\bar{x}$ =12.00, SD = 2.75), suggesting that most older participants possess a certain level of information awareness and behavioral motivation, showing tendencies toward both active and passive health information acquisition.

However, lower scores were noted in Environmental Factors ( $\bar{x}$ =8.32, SD = 2.75) and Role-Related Factors ( $\bar{x}$ =9.71, SD = 2.85), implying that older adults may face limitations in their external information environments—such as digital device accessibility and public information infrastructure—as well as in the roles they assume within family or social contexts. These constraints may adversely affect the manifestation of their information behavior.

In summary, the older population demonstrated significant variability in both structural capital and information behavior dimensions. These findings offer a solid empirical foundation for the subsequent correlation analyses and structural modeling phases of the study.

Summary statistics are presented for various dimensions associated with structural capital and health information behaviors, (e.g., Context of Information Need, Stress and Coping, Active/Passive Search). Higher scores indicate greater levels of the respective constructs.

### 5.2 Spearman correlation analysis

To further examine the associations between structural capital and the various dimensions of health information behavior, this study employed Spearman’s rank correlation coefficients to conduct bivariate correlation analyses between the five categories of capital and the 13 variables defined by Wilson’s information behavior model.

As illustrated in Figure 3, the results indicate multiple significant associations between structural capital and dimensions of information behavior, with cultural capital and psychological capital showing particularly strong correlations.

**Figure 2:**
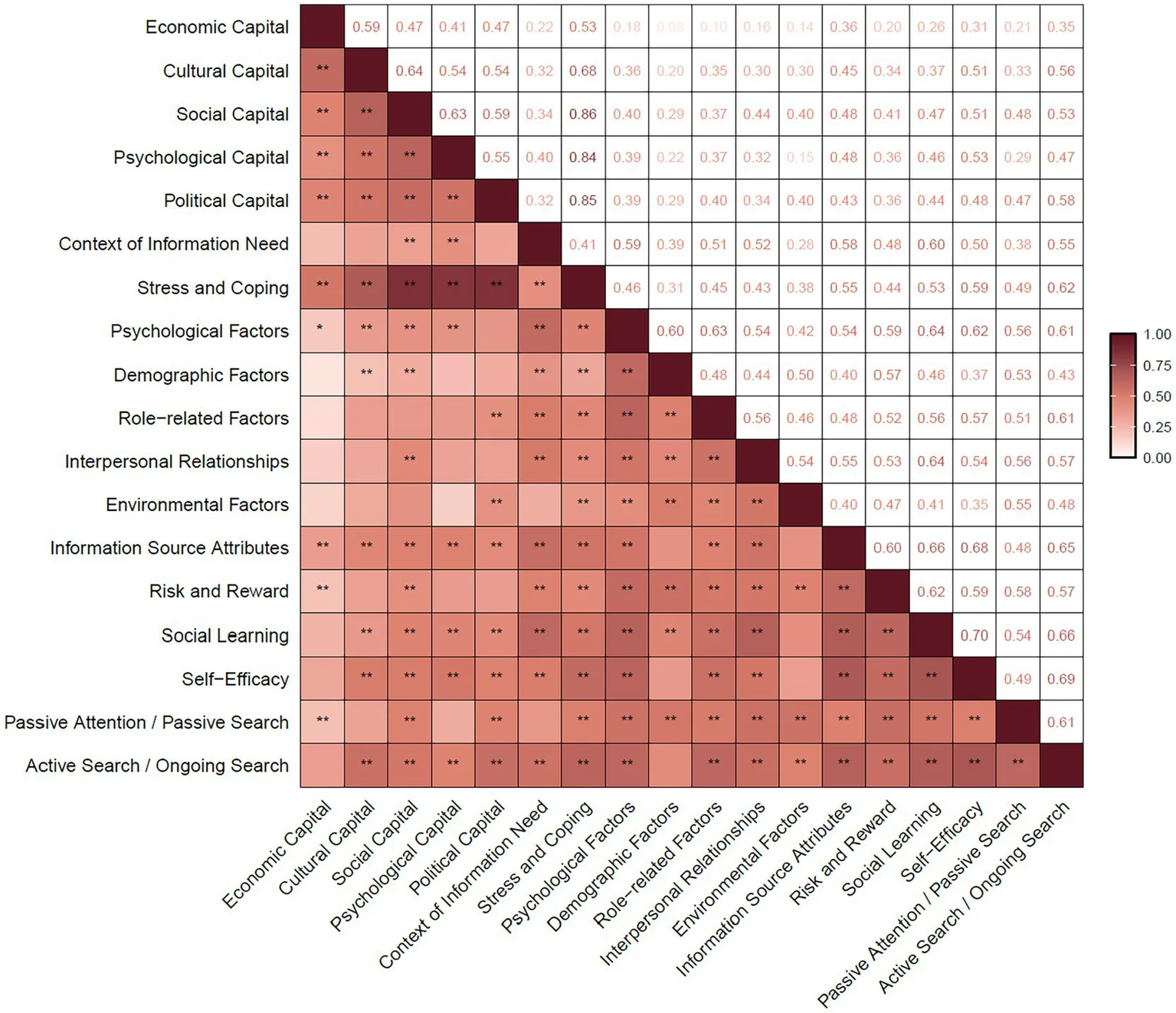
Spearman rank correlation coefficient heatmap between dimensions of structural capital and health information behavior. This heatmap illustrates the Spearman rank correlation coefficients among various dimensions of structural capital and health information behavior. Darker shades indicate stronger positive correlations. Statistical significance is denoted by asterisks, with *p*-values of less than 0.05 (*) and less than 0.01 (**). The matrix includes dimensions such as economic, cultural, social, psychological, and political capital, as well as contextual and psychological determinants of health information behavior, including search behavior, coping mechanisms, and self-efficacy.

Specifically, economic capital demonstrated moderate positive correlations with cultural capital ( $r_{s}=0.59$ ) and social capital ( $r_{s}=0.47$ ), suggesting a degree of linkage between financial conditions and both cultural literacy and access to social resources. However, the correlations between economic capital and behavioral variables such as demographic factors, role-related factors, and interpersonal relationships were weak and statistically insignificant, indicating that the direct association between economic capital and health information behavior is limited and may operate indirectly through other forms of capital.

Cultural capital showed strong positive correlations with both social capital ( $r_{s}=0.64$ ) and psychological capital ( $r_{s}=0.54$ ), indicating that higher educational attainment and cultural engagement are not only associated with more extensive social networks, but also closely linked to enhanced psychological resilience. These may be indirectly associated with behavioral patterns through individuals’ information acquisition ability and willingness.

Psychological capital emerged as the most significant factor. It exhibited a perfect positive correlation with stress adaptation capability ( $r_{s}=1.00$ ), underscoring the central role of emotional resilience and self-confidence in managing information-related stress. Additionally, self-efficacy was strongly correlated with active/sustained information search behavior ( $r_{s}=0.69$ ), indicating that older individuals with higher self-efficacy are more likely to engage in proactive health information seeking.

Overall, the correlation analysis provides initial evidence of associations between psychological, cultural, and social capital and health information behavior among older adults. These forms of capital showed interconnected patterns at the psychological and social levels. These findings provide an empirical basis for the subsequent Bayesian network analysis of conditional dependencies among the study variables.

### 5.3 Bayesian network modeling analysis

All 13 information behavior variables derived from Wilson’s model were included in the Bayesian network analysis. Four variables—Active/Ongoing Search, Information Source Attributes, Self-Efficacy, and Passive Attention/Search—were highlighted for interpretation as behavioral proxy indicators of vulnerability to the digital health information divide. The other dimensions of Wilson’s model remained part of the network to characterize the contextual, psychological, and social relationships surrounding these focal indicators. These indicators were not intended to directly measure the digital divide itself, but rather to capture behavioral manifestations associated with individuals’ ability to access, evaluate, and engage with digital health information. Active/Ongoing Search reflects the individual’s ability and willingness to proactively and continuously acquire digital health information; Information Source Attributes reflects preferences for reliable sources, the use of multiple sources, and access to useful and reliable information; Self-Efficacy reflects confidence in dealing with information technologies or complex health information; and Passive Attention/Search indicates greater reliance on incidental or unintentional exposure to information.

The selection of these four indicators was guided by Wilson’s information behavior model and the focus of this study on older adults’ digital health information behavior. Collectively, they reflect four complementary aspects: proactive information acquisition, preferences for and access to information sources, confidence in information seeking, and passive information exposure. These indicators were used to explore behavioral patterns potentially relevant to the digital health information divide, rather than to provide a comprehensive or direct measure of the divide itself. Passive attention/search should be interpreted in context, as incidental information exposure does not necessarily indicate disadvantage.

As shown in Figure 2, a total of 15 directed network chains were identified. These chains represent conditional dependencies within the sample and should not be interpreted as confirmed causal pathways:

(1) Cultural Capital → Psychological Capital → Self-Efficacy.
(2) Cultural Capital → Psychological Capital → Information Source Attributes.
(3) Cultural Capital → Social Capital → Demographic Factors → Social Learning → Active/Ongoing Search.
(4) Cultural Capital → Social Capital → Demographic Factors → Social Learning → Information Source Attributes.
(5) Cultural Capital → Political Capital → Active/Ongoing Search.
(6) Cultural Capital → Political Capital → Role-related Factors → Self-Efficacy.
(7) Cultural Capital → Risk and Reward → Passive Attention/Search.
(8) Psychological Capital → Political Capital → Active/Ongoing Search.
(9) Psychological Capital → Political Capital → Role-related Factor → Environmental Factors → Passive Attention/Search.
(10) Psychological Capital → Social Capital → Demographic Factors → Environmental Factors → Passive Attention/Search.
(11) Psychological Capital → Social Learning → Self-Efficacy.
(12) Psychological Capital → Social Learning → Interpersonal Relationships → Information Source Attributes.
(13) Political Capital → Economic Capital.
(14) Political Capital → Demographic Factors → Environmental Factors → Passive Attention/Search.
(15) Political Capital → Role-related Factor → Environmental Factors → Passive Attention/Search.

First, cultural capital occupied a highly connected position within the network. Lower cultural capital was linked to lower psychological capital and weaker self-efficacy (Chain 1), as well as a reduced ability to recognize information source attributes (Chain 2). Lower cultural capital was also connected with lower social capital, demographic and social learning variables, less frequent Active/Ongoing Search (Chain 3), and less accurate recognition of information source attributes (Chain 4). Other network chains connected cultural capital with Active/Ongoing Search through political capital (Chain 5), with self-efficacy through political capital and role-related factors (Chain 6), and with passive attention/search through risk–reward perceptions (Chain 7). These chains describe conditional-dependency patterns observed in the sample rather than confirmed temporal or causal sequences.

Second, psychological capital was connected with several variables relevant to the digital health information divide. Lower psychological capital was linked to political capital and less frequent Active/Ongoing Search (Chain 8), as well as to role-related and environmental factors associated with passive attention/search (Chain 9). It was also connected with environmental factors in a chain involving social capital and demographic factors (Chain 10), with self-efficacy through social learning (Chain 11), and with information source attributes through social learning and interpersonal relationships (Chain 12). These findings indicate that psychological capital occupies an intermediate network position across several conditional-dependency chains, but they do not establish mediation or causal effects.

Third, political capital was associated with several institutional, environmental, and behavioral variables in the network. It was linked to economic capital (Chain 13) and to passive attention/search through chains involving demographic and environmental factors (Chain 14) or role-related and environmental factors (Chain 15). These network connections suggest that political capital may be relevant to the institutional and environmental conditions surrounding older adults’ information behavior, although the cross-sectional data do not establish the direction or causal nature of these relationships.

In summary, the Bayesian network revealed interconnected conditional dependencies among the five forms of capital and the information behavior variables examined in this study. Lower cultural and psychological capital was associated with less proactive information behavior, while political and economic capital was connected with institutional and material resource conditions, and social capital was associated with external information-support relationships. Through their observed associations with Active/Ongoing Search, Passive Attention/Search, Self-Efficacy, and Information Source Attributes, the five forms of capital may be relevant to disparities in information access and use among older adults. However, these findings represent cross-sectional associations and network dependencies rather than confirmed causal mechanisms.

## 6 Discussion

### 6.1 Multi-pathway associations between structural capital, older adults’ health information behaviors, and the risk of the information divide

Addressing the core research question—how older adults’ economic, cultural, social, political, and psychological capital are associated with different stages of health information behaviors, and through these behavioral pathways potentially relate to their risk of the information divide—this study employs a Bayesian network–based systematic and networked analysis to characterize the multi-layered, cross-capital linkages and multi-pathway patterns embedded in the behavioral formation process. This approach not only transcends the limitations of prior research, which often emphasized single-capital effects or linear relationships, but also depicts the complex structure and cumulative associations underpinning older adults’ health information behaviors, thereby offering a multi-dimensional explanatory framework for the information divide. The results demonstrate that different forms of capital show distinct associations with behavioral variables while also forming interconnected patterns through cross-capital linkages—jointly corresponding to behavioral patterns at cognitive, motivational, institutional, and resource levels.

First, cultural capital emerges as the most complex and significant hub, showing direct links with older adults’ self-efficacy and ability to evaluate information source attributes, while also being indirectly connected with active/sustained search behaviors through social and political capital. This aligns with the nationwide empirical findings of Cui et al. (45), yet the novelty of this study lies in its first use of networked pathway analysis to explicitly identify cultural capital as the central node in cross-capital linkages. It functions not only as a key resource for informational competence but also as a platform supporting the joint roles of social and political capital; lower levels are associated with disruptions across multiple capital linkages.

Second, social capital exhibits cross-level dual functionality: on the one hand, it is directly linked to the evaluation of information source attributes and active/sustained search behaviors through interpersonal relationships and demographic factors, consistent with the systematic review by Grotto et al. (46), which emphasized the dual role of social capital in promoting both the frequency and quality of digital technology use. On the other hand, in cross-capital linkages, it may act as both a buffer and a catalyst—being associated with lower technology anxiety through support networks and with stronger links between psychological capital and information behavior. This finding extends beyond the conventional view of social capital as merely expanding information acquisition channels, highlighting its potential role in linking psychological resources to internalized capabilities and identifying it as a key co-factor associated with eHealth literacy.

Third, psychological capital plays a particularly prominent intermediary role within the network. Attributes such as self-confidence, optimism, and resilience are directly associated with self-efficacy and are also linked to the transformation of social capital into health information behaviors. This is consistent with Xu et al. (47) characterization of psychological capital as a core psychological resource for positive health behaviors, and aligns with Liao et al. (48) finding of an intermediary role between social capital and health-promoting behaviors. However, diverging from prior studies that examined single-path mediation, the Bayesian network in this study identifies a multi-pathway intermediary pattern for psychological capital, underscoring its potential systemic value as a cross-stage “bridge” within the capital–behavior–information divide chain.

Here, the intermediary role of psychological capital refers to its position along paths connecting other forms of capital with information behavior variables in the Bayesian network. No formal mediation analysis was conducted; therefore, these paths should not be interpreted as statistically established mediation effects.

Fourth, political capital is linked to the multi-channel availability of institutional resources. It is directly connected with active/sustained search behaviors and is also associated with passive attention/search through role-related and environmental factors, thus reflecting the external support environment. This resonates with De Luca et al. (49) findings that public health systems not only improve healthcare accessibility but also foster the accumulation of both social and political capital among older adults. Our study further identifies interconnections among political capital, social capital, and cultural capital—suggesting that institutional guarantees should be viewed as embedded components rather than isolated influences.

Lastly, although economic capital appears primarily in indirect pathways to behavioral variables, its foundational role is undeniable. As a material prerequisite for information acquisition, economic capital is associated with access to resources and the diversity of information sources. Ghahfarokhi et al. (50) noted that inadequate economic conditions often limit older adults’ access to technology and training opportunities, thereby reducing their level of digital health literacy. This study identifies a pathway consistent with the structural pattern of “economic capital → access → information behavior,” suggesting that intervention strategies should prioritize enhancing access and training for groups with limited economic capital to prevent the weakening of the entire behavioral chain.

In summary, the five forms of structural capital are linked through direct, indirect, and cross-capital pathways to distinct stages of older adults’ health information behaviors and to their risk of the information divide. Among these, cultural and psychological capital serve as the central hubs of the behavioral network; social and political capital jointly correspond to institutional and relational safeguards; and economic capital underpins the material foundation. This networked, multi-dimensional association model not only deepens the understanding of the patterns related to the digital health information divide, but also offers a precise pathway mapping for targeted interventions addressing capital-specific deficiencies.

### 6.2 Governance pathways for the older adult digital health information divide based on multi-capital network analysis

The governance of the older adult digital health information divide should move beyond the traditional “single-capital–single-stage” intervention model, toward a systemic and synergistic governance framework structured along the logical chain of capital networks–behavioral pathways–risk mitigation. This approach emphasizes the intrinsic integration of multi-capital synergy, targeted intervention, and institutional safeguards, which can be advanced through the following three interrelated dimensions.

#### 6.2.1 Systemic advancement through capital empowerment

Bayesian network analysis indicates that although economic capital is linked predominantly through indirect pathways to behavioral variables, its foundational role in facilitating information access, device acquisition, and diversification of access channels constitutes a prerequisite for the effective mobilization of other capitals. Cultural capital, with the highest betweenness centrality in the network, is directly associated with information discernment and self-efficacy and is also indirectly linked to active/sustained search behaviors through psychological capital. Psychological capital occupies an intermediary position across multiple pathways, connecting social and cultural capital with behavioral variables. Social capital and political capital, respectively, correspond to interpersonal support and institutional guarantees, with notable interconnections across the network.

Accordingly, governance should construct a full-chain empowerment framework progressing from material access–capacity enhancement–psychological motivation–social support–institutional assurance, thereby promoting dynamic coupling and positive feedback among capitals. For instance, deploying publicly accessible digital health service terminals with minimal entry barriers in community settings, implementing tiered and group-specific cultural capital training, integrating emotional empowerment and psychological interventions to strengthen psychological capital, leveraging community and family networks to bolster social capital, and embedding sustained political capital support within public policy together constitute a systemic advancement model for cross-capital synergy.

#### 6.2.2 Precision interventions anchored in behavioral pathways

Findings indicate clear heterogeneity in the pathways linking different forms of capital to behavior: cultural capital is connected with self-efficacy and information source evaluation through psychological capital; social capital is indirectly associated with information discernment through technological anxiety and trust; and political capital is directly linked to active/sustained search and is also associated with passive attention and passive search through environmental and role-related factors.

Therefore, governance should deploy targeted interventions around critical behavioral nodes. For active/sustained search, personalized recommendation algorithms and context-specific guidance can be leveraged to reinforce advanced information behaviors driven by cultural capital. For passive attention/search, optimizing the credibility and presentation of pushed content can reduce psychological resistance and cognitive load. For self-efficacy and information discernment, AI-enabled credible information evaluation tools, coupled with emotional support and trust endorsement mechanisms, can enhance the conversion rate of psychological capital into effective behaviors. Such a behavior-chain-based intervention model uses key nodes identified by Bayesian network analysis as strategic entry points, enabling optimal allocation of intervention resources and maximization of impact.

#### 6.2.3 Institutionalized and networked long-term mechanisms

In this study’s network analysis, political capital is not only directly connected with behavior but also exhibits notable interconnections with cultural and social capital, suggesting that institutional safeguards may act as “catalysts” within the multi-capital network. Therefore, governance should integrate the enhancement of older adult digital health literacy and the narrowing of the health information divide into a unified national policy framework encompassing public health, digital inclusion, and social protection. It is essential to foster regularized collaboration among healthcare institutions, community organizations, information platforms, and academic bodies, underpinned by institutionalized cross-sector coordination mechanisms, dynamic monitoring and evaluation systems, and data-driven iterative strategies. This will enable the formation of a governance loop of real-time monitoring–precision optimization–sustained implementation, ensuring that interventions remain adaptive and sustainable across diverse capital and behavioral nodes. Furthermore, establishing a nationwide “Older Adult Digital Health Capital Enhancement Index” monitoring system could provide quantitative benchmarks for policy evaluation and resource allocation.

In sum, this study identifies multi-pathway linkages between five categories of structural capital and the health information behavior chain and innovatively applies Bayesian network analysis to the governance of the older adult digital health information divide. By revealing the structural connections between cross-capital linkages and behavioral nodes, the study proposes a systematic, precise, and sustainable governance pathway. The integration of theory and method presented here offers a transferable academic model and practical framework for implementing governance strategies across diverse cultural and institutional contexts.

## 7 Conclusion

Grounded in structural poverty theory and Wilson’s information behavior model, this study employs Bayesian network analysis to map the multi-pathway linkages between economic, cultural, social, political, and psychological capital and different stages of older adult health information behavior. The results indicate that lower levels of specific forms of capital are associated with disparities in information acquisition and utilization, including lower self-efficacy, a reduced ability to evaluate information sources, and less frequent active/sustained search, which may contribute to the manifestation and persistence of the digital health information divide. Among the capitals, cultural capital occupies a pivotal hub position; psychological capital occupies an intermediate network position connecting multiple capital and behavioral variables; social and political capital correspond to external support and institutional safeguards; and economic capital reflects the foundational conditions for resource access.

On this basis, the study proposes three categories of governance strategies—capital empowerment, precision intervention along behavioral pathways, and institution–ecosystem synergies—to enhance key capital levels, optimize the health information behavior chain, and build a sustainable enabling environment, thereby providing both a theoretical foundation and practical pathways for narrowing the older adult digital health information divide.

## 8 Limitations and future directions

Despite its theoretical integration and methodological innovation, this study has four notable limitations. First, the sample was drawn from a specific geographic region, which effectively reflects the characteristics of the study population but may limit generalizability. Future research should conduct cross-regional comparative analyses that account for diverse socio-economic and cultural contexts. Second, this study used cross-sectional data primarily derived from self-reported questionnaires. Although Bayesian network analysis effectively reveals multi-pathway conditional dependencies among capitals and information behavior variables, the cross-sectional design cannot establish temporal precedence or definitive causal relationships. Therefore, the directed edges in the Bayesian network should be interpreted as probabilistic dependencies and potential pathways rather than confirmed causal effects. Future studies could incorporate longitudinal tracking and multi-source behavioral data to verify the direction and stability of these pathways and capture their dynamic evolution.

Third, Active/Ongoing Search, Information Source Attributes, Self-Efficacy, and Passive Attention/Search were used as behavioral proxy indicators relevant to the digital health information divide rather than as direct or comprehensive measures of the divide itself. These variables capture selected aspects of information acquisition, source evaluation, confidence, and passive exposure, but do not fully represent other dimensions of the divide, such as actual digital access, technology use, or information-use outcomes. This may narrow the interpretation of the findings and limit their generalizability to the broader construct of the digital health information divide. Fourth, the average questionnaire scores were categorized into low, medium, and high states for the discrete Bayesian network. Although this facilitated the estimation and interpretation of conditional probabilities, categorization may reduce variability, classify participants with similar scores near the cutoffs into different groups, and affect the learned network structure and probability estimates. The findings may therefore be sensitive to the selected cutoffs and may not be directly generalizable to samples with different score distributions. Future studies should examine alternative cutoffs and continuous or mixed-variable Bayesian network models to assess the robustness of the findings.

Subsequent research should further refine the model structure using more diverse samples and mixed data sources, exploring the long-term patterns of capital evolution and information behavior change, so as to provide more universal and dynamic theoretical support for governing the older adult digital health information divide.

## Data Availability

The datasets presented in this study can be found in online repositories. The names of the repository/repositories and accession number(s) can be found at: The data can be obtained through the following link address: https://doi.org/10.57760/sciencedb.28114.
