# Supplementary material for: Study on the formation mechanism of the digital health information divide and capital-driven pathways among the older adults
Source: Front Public Health. 2026 Oct 29;14:1913689. doi: 10.3389/fpubh.2026.1913689 (PMC13758375; doi:10.3389/fpubh.2026.1913689)
Supplement: Supplementary file 1 [file Supplementary_file_1.docx]

**Dear Participant,**

Hello! Thank you very much for participating in this questionnaire survey on the "Governance Strategies for the Digital Health Information Divide Among the Elderly in the Digital Age." This study has been approved by the Ethics Committee of Nanjing Medical University. The research aims to explore the current status, challenges, and influencing factors in the process of acquiring digital health information by the elderly, especially the impact of economic and cultural capital on information-seeking behaviors. Through your responses, we will gain a better understanding of how elderly individuals acquire health information in the digital age and how to formulate more effective health information support policies.

Please rest assured that all responses are anonymous, and your personal information and answers will be strictly confidential. All survey data will be used for academic research purposes only and will not disclose any of your personal privacy. This survey will take approximately 10 minutes to complete, and each of your responses is very important to us. Please answer truthfully based on your actual situation. Your opinions will provide valuable references for this study.

Thank you for your participation and support!

**1. In the past month, when you encountered health issues, did you feel an urgent need to obtain health information to resolve the issue?**

- Strongly Disagree
- Disagree
- Neutral
- Agree
- Strongly Agree

**2. When facing health issues, do you have multiple types of information needs? (e.g., treatment methods, preventive measures, health lifestyle advice, etc.)**

- Not needed at all
- Slightly needed
- Neutral
- Quite needed
- Very needed

**3. Do you believe that obtaining information is crucial for making decisions when facing health problems?**

- Strongly Disagree
- Disagree
- Neutral
- Agree
- Strongly Agree

**4. When experiencing psychological stress due to health issues, do you tend to actively search for relevant health information to relieve stress?**

- Strongly Disagree
- Disagree
- Neutral
- Agree
- Strongly Agree

**5. When experiencing emotional pressure from health issues, do you feel that seeking health information helps alleviate anxiety and discomfort?**

- Strongly Disagree
- Disagree
- Neutral
- Agree
- Strongly Agree

**6. When facing health issues, do you seek health information by consulting friends or family to relieve psychological stress?**

- Never seek
- Rarely seek
- Occasionally seek
- Often seek
- Always seek

**7. When looking for health information, do you believe you can find useful information?**

- Strongly Disbelieve
- Rarely believe
- Neutral
- Quite believe
- Strongly believe

**8. When facing health issues, are you easily discouraged from actively seeking relevant information due to emotional fluctuations?**

- Strongly Disagree
- Disagree
- Neutral
- Agree
- Strongly Agree

**9. When you encounter health problems, do you persist in seeking information through multiple channels rather than giving up easily?**

- Never persist
- Rarely persist
- Sometimes persist
- Often persist
- Always persist

**10. When facing health issues, do you think your age affects your ability to acquire health information?**

- Strongly Disagree
- Disagree
- Neutral
- Agree
- Strongly Agree

**11. Does your educational background help you better understand and use health information?**

- Strongly Disagree
- Disagree
- Neutral
- Agree
- Strongly Agree

**12. Does your economic situation influence how you acquire health information (e.g., whether you can afford paid health information or consultation services)?**

- Not at all
- Rarely
- Sometimes
- Often
- Always

**13. Do you feel responsible for health-related decisions in your family, prompting you to actively seek health information?**

- Never responsible
- Rarely responsible
- Sometimes responsible
- Often responsible
- Always responsible

**14. Does your work require you to understand and use health-related information, motivating you to actively search for information?**

- Never required
- Rarely required
- Sometimes required
- Often required
- Always required

**15. In society, do you feel that you are regarded as a "provider" of health knowledge, making you more active in seeking and sharing health information?**

- Strongly Disagree
- Disagree
- Neutral
- Agree
- Strongly Agree

**16. When you encounter health issues, do you seek information or advice from your family?**

- Never seek
- Rarely seek
- Occasionally seek
- Often seek
- Always seek

**17. When you encounter health issues, do you seek related health information from friends or colleagues?**

- Never seek
- Rarely seek
- Occasionally seek
- Often seek
- Always seek

**18. Does your social circle (e.g., friends, colleagues) positively influence your health information-seeking behavior?**

- No influence
- Slight influence
- Sometimes has an influence
- Often has an influence
- Always has an influence

**19. Do you think the convenience of internet access affects how you acquire health information?**

- No effect
- Slight effect
- Somewhat affects
- Often affects
- Always affects

**20. Have you participated in any community-organized health-related activities and gained health information from them?**

- Never participate
- Rarely participate
- Occasionally participate
- Often participate
- Always participate

**21. Does your living environment affect the ways you acquire health information (e.g., availability of sufficient health information resources or support)?**

- No effect
- Slight effect
- Somewhat affects
- Often affects
- Always affects

**22. Do you tend to choose reliable health information sources (e.g., professional medical websites or doctors) to obtain information?**

- Strongly Disagree
- Disagree
- Neutral
- Agree
- Strongly Agree

**23. Do you tend to choose multiple sources of information to obtain health information (e.g., internet, books, doctors, family, etc.)?**

- Never choose
- Rarely choose
- Occasionally choose
- Often choose
- Always choose

**24. Is it easy for you to access what you consider reliable and useful health information sources (e.g., medical institutions, the internet)?**

- Not easy at all
- Rarely easy
- Neutral
- Often easy
- Always easy

**25. When searching for health information, do you feel there are risks involved (e.g., misleading or unreliable information)?**

- Strongly Disagree
- Disagree
- Neutral
- Agree
- Strongly Agree

**26. When facing health problems, do you believe searching for health information can bring significant benefits (e.g., better health decisions, faster treatment, etc.)?**

- Strongly Disagree
- Disagree
- Neutral
- Agree
- Strongly Agree

**27. When you search for health information, do you evaluate the cost of searching (e.g., time, money, effort) against the potential benefit of the information?**

- Never consider
- Rarely consider
- Sometimes consider
- Often consider
- Always consider

**28. When encountering health problems, do you refer to others' experiences (e.g., family, friends, doctors) to decide whether to search for information?**

- Never refer
- Rarely refer
- Occasionally refer
- Often refer
- Always refer

**29. Do you think advice and support from family or friends motivate you to actively seek health information?**

- Strongly Disagree
- Disagree
- Neutral
- Agree
- Strongly Agree

**30. When you see others (e.g., family, friends, celebrities) achieve positive outcomes through searching for health information, are you more inclined to actively seek information yourself?**

- Strongly Disagree
- Disagree
- Neutral
- Agree
- Strongly Agree

**31. When you need to search for health information, do you feel you have enough ability to find accurate and useful information?**

- Strongly Disagree
- Disagree
- Neutral
- Agree
- Strongly Agree

**32. In the process of information-seeking, do you believe you can overcome technical difficulties (e.g., using the internet, finding reliable health information sources)?**

- Strongly Disbelieve
- Rarely believe
- Neutral
- Quite believe
- Strongly believe

**33. When facing health problems, do you search for health information because you feel confident about the information-seeking process?**

- Strongly Disagree
- Disagree
- Neutral
- Agree
- Strongly Agree

**34. Have you unintentionally encountered health-related information (e.g., from advertisements, social media recommendations, etc.)?**

- Never
- Rarely
- Occasionally
- Often
- Always

**35. Have you received health-related information via social media, email, or other platforms without actively seeking it?**

- Never
- Rarely
- Occasionally
- Often
- Always

**36. When you need health information, do you tend to rely on family or friends to provide relevant information rather than actively seeking it yourself?**

- Never rely
- Rarely rely
- Sometimes rely
- Often rely
- Always rely

**37. Do you usually receive health information through friends, family, or social networks rather than actively searching for it?**

- Never receive
- Rarely receive
- Occasionally receive
- Often receive
- Always receive

**38. When you encounter health problems, do you actively search for related health information on the internet?**

- Never actively
- Rarely actively
- Occasionally actively
- Often actively
- Always actively

**39. When you need health information, do you actively consult doctors or other health experts for accurate information?**

- Never actively
- Rarely actively
- Occasionally actively
- Often actively
- Always actively

**40. Do you regularly or continuously seek health-related information, such as by subscribing to health websites or regularly reading health news?**

- Never regularly
- Rarely regularly
- Occasionally regularly
- Often regularly
- Always regularly

**41. When you encounter health problems, do you continue to monitor related information until the issue is resolved?**

- Never monitor
- Rarely monitor
- Occasionally monitor
- Often monitor
- Always monitor

**42. What is your monthly income approximately?**

- Less than 3000 RMB
- 3000-5000 RMB
- 5000-8000 RMB
- 8000-12000 RMB
- Above 12000 RMB

**43. Do you own any of the following fixed assets (e.g., real estate, car, etc.)?**

- None
- One fixed asset (e.g., only real estate)
- Two fixed assets (e.g., real estate and car)
- Three fixed assets (e.g., real estate, car, savings)
- Four or more fixed assets

**44. In the past month, what was your total expenditure on daily consumption (e.g., food, entertainment, travel, etc.)?**

- 0-500 RMB
- 501-1000 RMB
- 1001-2000 RMB
- 2001-5000 RMB
- Above 5000 RMB

**45. Do you think your economic situation is sufficient to meet all your daily needs (including basic living, entertainment, savings, etc.)?**

- Completely insufficient, significant financial pressure
- Slightly insufficient, frequent financial pressure
- Adequate, occasional financial pressure
- Sufficient, able to meet daily needs and some extra expenses
- Very abundant, very good economic situation

**46. What is your highest level of education?**

- Elementary school or below
- Junior high school
- High school or vocational school
- College or university
- Master's degree or higher

**47. Do you regularly participate in cultural activities (e.g., reading books, magazines, visiting museums, attending lectures, etc.)?**

- Never participate
- Occasionally participate
- Sometimes participate
- Often participate
- Always participate

**48. Are you proficient in using the internet to search and acquire information?**

- Completely unskilled
- Slightly unskilled
- Neutral
- Quite skilled
- Very skilled

**49. Do you regularly update your knowledge in your professional field or areas of personal interest through reading, learning, etc.?**

- Never learn
- Occasionally learn
- Sometimes learn
- Often learn
- Always learn

**50. Do you have a stable social network (e.g., friends, colleagues, neighbors) that can offer support?**

- None
- Rarely
- Some, but not often in contact
- Stable, occasionally supportive
- Stable, frequently supportive

**51. When facing difficulties in life, can you get support or help from family or friends?**

- Never
- Rarely
- Occasionally
- Often
- Always

**52. Do you regularly participate in activities in your community (e.g., community gatherings, volunteer services, other community-organized activities)?**

- Never participate
- Occasionally participate
- Sometimes participate
- Often participate
- Always participate

**53. Do you maintain close interaction and contact with people in your social circle (e.g., friends, neighbors, colleagues)?**

- No interaction
- Occasionally interact
- Occasionally, sometimes interact
- Often interact
- Always maintain interaction

**54. Do you believe you can overcome difficulties and succeed when facing challenges?**

- Strongly disbelieve
- Rarely believe
- Neutral
- Usually believe
- Strongly believe

**55. When encountering difficulties, can you recover quickly and move on?**

- Cannot recover
- Difficult to recover
- Sometimes recover
- Usually recover
- Always recover

**56. In the past month, have you felt happy and emotionally stable?**

- Never
- Rarely
- Occasionally
- Often
- Always

**57. When facing life pressures, can you manage effectively and stay calm?**

- Cannot manage
- Difficult to manage
- Sometimes manage
- Usually manage
- Always manage

**58. Do you believe you can equally access the social resources you need in life (e.g., education, healthcare, housing, etc.)?**

- Strongly disagree
- Disagree
- Neutral
- Agree
- Strongly agree

**59. Do you feel you have some level of participation and voice in social and political decisions?**

- Never
- Rarely
- Sometimes
- Often
- Always

**60. Do you think social resources (e.g., welfare, employment opportunities, etc.) are fairly distributed in your social group?**

- Totally unfair
- Slightly unfair
- Neutral
- Fair
- Very fair

**61. In the area where you live, can you access timely and clear information about government policies and social services?**

- Never
- Rarely
- Occasionally
- Often
- Always
