# Supplementary material for: Study on the formation mechanism of the digital health information divide and capital-driven pathways among the older adults
Source: Front Public Health. 2026 Oct 29;14:1913689. doi: 10.3389/fpubh.2026.1913689 (PMC13758375; doi:10.3389/fpubh.2026.1913689)
Supplement: Supplementary file 2 [file Supplementary_file_2.docx]

Supplementary Table S1

**Mapping of questionnaire items to study variables**

The 61 questionnaire items map to 13 information behavior variables and five capital variables. Item numbers follow the administered Chinese questionnaire. English content summaries below describe the items; they are not verbatim translations.

| **Variable** | **Items** | **Item content summaries** |
| --- | --- | --- |
| Context of Information Need | 1–3 | 1. Urgent need for health information when facing a health problem in the past month. 2. Need for multiple types of health information. 3. Perceived importance of health information for decision-making. |
| Stress and Coping | 4–6 | 4. Seeking health information to reduce health-related psychological stress. 5. Perceived relief of anxiety and unease through seeking health information. 6. Consulting friends or family for health information and stress relief. |
| Psychological Factors | 7–9 | 7. Belief in being able to find useful health information. 8. Giving up active information seeking because of emotional fluctuations. 9. Persistence in obtaining information through multiple channels. |
| Demographic Factors | 10–12 | 10. Perceived influence of age on the ability to obtain health information. 11. Perceived help from educational background in understanding and using health information. 12. Perceived influence of economic circumstances on information access channels. |
| Role-related Factors | 13–15 | 13. Family responsibility for health-related decisions and motivation to seek information. 14. Work requirements for health information and motivation to seek it. 15. Being regarded as a provider of health knowledge and motivation to seek and share information. |
| Interpersonal Relationships | 16–18 | 16. Seeking information or advice from family. 17. Seeking health information from friends or colleagues. 18. Positive influence of the social circle on health information seeking. |

Supplementary Table S1 continued

**Mapping of questionnaire items to study variables**

| **Variable** | **Items** | **Item content summaries** |
| --- | --- | --- |
| Environmental Factors | 19–21 | 19. Perceived influence of internet accessibility on ways of obtaining health information. 20. Participation in community health activities and information gained through them. 21. Perceived influence of the residential environment on information access channels. |
| Information Source Attributes | 22–24 | 22. Preference for reliable health information sources. 23. Selection of multiple health information sources. 24. Ease of access to sources perceived as reliable and useful. |
| Risk and Reward | 25–27 | 25. Perceived risks during health information seeking. 26. Perceived benefits of seeking health information. 27. Consideration of search costs and potential information benefits. |
| Social Learning | 28–30 | 28. Consulting others’ experiences when deciding whether to seek information. 29. Motivation to seek information from family or friends’ advice and support. 30. Motivation to seek information after observing others’ positive outcomes. |
| Self-Efficacy | 31–33 | 31. Confidence in being able to find accurate and useful health information. 32. Confidence in overcoming technical difficulties in information seeking. 33. Active health information seeking motivated by confidence in the search process. |
| Passive Attention / Passive Search | 34–37 | 34. Incidental exposure to health information. 35. Receiving health information through digital platforms without actively seeking it. 36. Reliance on family or friends for information rather than active searching. 37. Receiving information through family, friends, or social networks rather than active searching. |

Supplementary Table S1 continued

**Mapping of questionnaire items to study variables**

| **Variable** | **Items** | **Item content summaries** |
| --- | --- | --- |
| Active Search / Ongoing Search | 38–41 | 38. Active internet searching when facing health problems. 39. Active consultation with doctors or other health experts. 40. Regular or ongoing health information seeking. 41. Continued attention to relevant information until a health problem is resolved. |
| Economic Capital | 42–45 | 42. Monthly income category. 43. Reported asset ownership category. 44. Monthly expenditure on daily consumption. 45. Perceived adequacy of economic resources for daily needs. |
| Cultural Capital | 46–49 | 46. Highest educational attainment. 47. Frequency of participation in cultural activities. 48. Self-reported proficiency in searching for and obtaining information online. 49. Ongoing learning and updating of knowledge. |
| Social Capital | 50–53 | 50. Availability of a stable social network offering support. 51. Access to help from relatives and friends during difficulties. 52. Frequency of participation in community activities. 53. Frequency of interaction with friends, neighbors, or colleagues. |
| Psychological Capital | 54–57 | 54. Confidence in overcoming challenges and succeeding. 55. Ability to recover from difficulties and continue. 56. Positive mood and emotional stability during the past month. 57. Ability to manage stress and remain calm. |
| Political Capital | 58–61 | 58. Perceived equal access to needed social resources. 59. Perceived participation and voice in social and political decisions. 60. Perceived fairness of resource distribution within the social group. 61. Access to timely and clear information about government policies and social services. |

Note. Each variable has three or four items. Each item offers five ordered response options, with anchors varying by item (e.g., agreement, frequency, confidence, income, or education). Demographic Factors measures perceived influences of demographic circumstances rather than recording age, education, and income directly.
